# Sleep–Wake Cycle and EEG–Based Biomarkers during Late Neonate to Adult Transition

**DOI:** 10.3390/brainsci11030298

**Published:** 2021-02-27

**Authors:** Miguel A. Herrero, Rebeca Gallego, Milagros Ramos, Juan Manuel Lopez, Guillermo de Arcas, Daniel Gonzalez-Nieto

**Affiliations:** 1 Center for Biomedical Technology (CTB), Universidad Politécnica de Madrid, 28223 Pozuelo de Alarcón, Spain; ma.herrero@upm.es (M.A.H.); rebegall@ucm.es (R.G.); milagros.ramos@upm.es (M.R.); 2 Departamento de Tecnología Fotónica y Bioingeniería, ETSI Telecomunicaciones, Universidad Politécnica de Madrid, 28040 Madrid, Spain; 3 Instrumentation and Applied Acoustics Research Group (I2A2), ETSI Topografía, Universidad Politécnica de Madrid, 28031 Madrid, Spain; juanmanuel.lopez@upm.es; 4 Laboratorio de Neuroacústica, Universidad Politécnica de Madrid, 28031 Madrid, Spain; 5 Biomedical Research Networking Center in Bioengineering Biomaterials and Nanomedicine (CIBER-BBN), 28029 Madrid, Spain; 6 Departamento de Ingeniería Telemática y Electrónica, ETSI Sistemas de Telecomunicación, Universidad Politécnica de Madrid, 28031 Madrid, Spain; 7 Departamento de Ingeniería Mecánica, ETSI Industriales, Universidad Politécnica de Madrid, 28006 Madrid, Spain

**Keywords:** brain, sleep disorders, electroencephalogram, biomarkers, neurodegeneration

## Abstract

During the transition from neonate to adulthood, brain maturation establishes coherence between behavioral states—wakefulness, non-rapid eye movement, and rapid eye movement sleep. In animal models few studies have characterized and analyzed cerebral rhythms and the sleep–wake cycle in early ages, in relation to adulthood. Since the analysis of sleep in early ages can be used as a predictive model of brain development and the subsequent emergence of neural disturbances in adults, we performed a study on late neonatal mice, an age not previously characterized. We acquired longitudinal 24 h electroencephalogram and electromyogram recordings and performed time and spectral analyses. We compared both age groups and found that late neonates: (i) spent more time in wakefulness and less time in non-rapid eye movement sleep, (ii) showed an increased relative band power in delta, which, however, reduced in theta during each behavioral state, (iii) showed a reduced relative band power in beta during wakefulness and non-rapid eye movement sleep, and (iv) manifested an increased total power over all frequencies. The data presented here might have implications expanding our knowledge of cerebral rhythms in early ages for identification of potential biomarkers in preclinical models of neurodegeneration.

## 1. Introduction

Sleep is a state of cerebral activity that is regulated by different brain structures, including the hypothalamus, brain stem and basal ganglia. The time and depth of sleep is influenced by the circadian rhythm and the duration of previous wakefulness. Studies in rodents have shown that sleep facilitates neural maturation and prevents apoptosis in developing brains [1,2]. Brain maturation establishes coherence between behavioral states—wakefulness, non-rapid eye movement (NREM), and rapid eye movement (REM) sleep. During development, the time spent in REM sleep slowly decreases, while time spent in NREM sleep increases [3,4,5].

The involvement of NREM and REM sleep in human brain physiology can be inferred by the comorbidity of sleep disorders (SDs) in a great majority of cerebrovascular and neurodegenerative disorders (NDs). For example, in Alzheimer’s disease (AD), the most common cause of dementia in older adults, sleep is highly fragmented, with a circadian disruption leading to daytime hypersomnia and nighttime insomnia. In Parkinson’s disease (PD), it is known that neuronal death in the substantia nigra pars compacta (SNpc) is linked to a reduced amount of time spent in REM sleep [6], which is accepted to be a supportive diagnostic criterion, and increased sleep fragmentation has also been reported [6,7,8]. Abnormal cerebral rhythms have been found in a variety of NDs. For example, AD mouse models exhibit a decrease in low-frequency bands (δ and θ bands) and an increase in high-frequency bands (α and β) [9]. A recent longitudinal study on PD patients suggests increased θ band power and decreased dominant (peak) frequency as biomarkers for disease progression, given their correlation with cognitive decline.

Sleep disorders might predict the functional outcome in several pathologies, but early EEG/sleep abnormalities might be associated with the initial impairment of neural networks before clinical signs manifest [7,10]. Poor linguistic ability in early life was translated into poor cognitive function and dementia six decades later [11]. This was hypothetically related to the appearance of subtle neuropathologic changes in early life that conditioned the brain’s evolution towards dementia and AD. It is reasonable to assume that features extracted from brain activity in neurodegenerative rodent models might be relevant as diagnostic biomarkers for neurodegeneration and predictive projection at clinical scenario, overall if they are studied at early, asymptomatic stages. However, our knowledge of cerebral rhythms at early ages in non-modified rodents in relation with adult age is still limited. A better knowledge concerning the homeostatic EEG rhythms and light/dark-induced sleep–wake cycle at these ages might provide “signatures” during the neonatal–adult transition and help us to understand how specific brain pathologies might impair these homeostatic signals.

## 2. Materials and Methods

### 2.1. Animals

Six neonatal and eleven adult male C57BL/6 mice were housed under 12-h light/dark phases with food and water ad libitum. Mice were manipulated by male and female experimenters. All experiments were conducted with the approval of the Research Ethics Committee at the Universidad Politécnica de Madrid, (Madrid, Spain), and complied with local and national ethical and legal regulations regarding the use of mice in research.

### 2.2. Surgery

Neonatal mice received surgery for placement of EEG electrodes at the age of 30 days, and adult mice at the age of 3 months. Starting from the day of surgery, each animal was housed in a separate cage. Surgery was performed under anesthesia (ketamine, an NMDA receptor antagonist; Imalgene, Merial, Lyon, France, 80 mg/kg, i.p.) and tranquilizer (xylazine, alpha-2 adrenergic receptor agonist; Rompun, Bayer, Leverkusen, Germany, 10 mg/kg, i.p.). Once pedal withdraw reflex ceased, the skin was prepared with iodopovidone (Betadine, Avrio Health L.P., Stamford, CT, USA) and alcohol wipes. After a small midline vertical incision to expose the skull was made, small craniotomies were performed at electrode locations (specified below) using a dental drill (NSK, Tokyo, Japan, MIO-230). In total, five electrodes were implanted. Two stainless steel screw electrodes (Plastics One Inc., Roanoke, VA, USA) were implanted into the skull over the left and right frontal cortex (AP + 0.5 mm; L ± 2.0 mm from bregma) as surface EEG recording electrodes. One stainless steel screw electrode was implanted in the neck muscle as an EMG recording electrode. Two additional stainless steel screw electrodes were placed over the left and right parietal cortex (AP − 1.0 mm; L ± 2.0 mm from bregma) as ground and reference electrodes, respectively. Once the skull dried, the coated portion of the wires was secured to the skull using a gel glue (Loctite 454, Henkel, Düsseldorf, Germany) and was covered with dental cement (Inlay Pattern Resin Powder and Liquid. DuraLay, Lancashire, UK,). The wires were connected to the header (MS363, Plastics One Inc., Roanoke, VA, USA), which was angled towards the ceiling. Mice received buprenorphine (0.05 mg/kg) and recovered in a warmed chamber for one hour prior to returning to a standard housing environment.

### 2.3. EEG and EMG Recordings

Longitudinal EEG and EMG recordings were acquired in 24 h sessions and split into 12 h light/dark phases. Mice were transferred to the recording room one week prior to recording sessions to allow for recovery and habituation. Freely moving mice were placed in a circular cage with a standard housing environment with food and water ad libitum. A flexible cable was attached to the header (Plastics One Inc., Roanoke, VA, USA) on the head and connected to single-channel AC amplifiers (78D, Grass, West Warwick, RI, USA,) that included 50 Hz. notch filter for power line frequency removal. Bilateral cortical EEG signals and EMG signal were acquired using the right parietal electrode as a reference and the signals amplified at 8.000 with 0.3–100 Hz. (EEG) and 30–100 Hz. (EMG) band-pass filters (CyberAmp, 380, Axon Instruments, San Jose, CA, USA). Signals were analog-to-digital converted (National Instruments, Austin, TX, USA, BNC-2090A) at a sampling frequency of 500 Hz. and recorded with LabVIEW Biomedical Toolkit software (National Instruments, Austin, TX, USA). 

### 2.4. Sleep–Wake Analysis

Behavioral states (wakefulness, NREM, and REM sleep) were determined by the analysis of EEG and EMG recordings with AccuSleep [12], a mouse-specific, semi-automated sleep–wake scoring algorithm written in MATLAB (R2018b, The MathWorks, Natick, MA, USA). The epoch length was set to 2.5 s. First, 24 h signal integrity and cleanliness (lack of noise) was visually confirmed. Second, some epochs of each state were scored manually by an expert scorer according to standard criteria. Third, these epochs were used for subject-specific calibration, namely the computation of mixture z-scoring parameters. Fourth, we performed the automated classification of behavioral states with the sleep scoring artificial neural network provided with the package, which was trained and validated on data scored at the same epoch length. Fifth, we revised manually the classification. For spectral analysis, we calculated the power within the 0–250 Hz. frequencies with 375 bin size (the frequency resolution of each spectral line equals 0.666 Hz.).

### 2.5. Statistical Analysis

Data shown as mean ± SEM. An unpaired t-test was performed to examine significant differences between neonatal and adult mice in power. Two-way analyses of variance (ANOVA) were performed to examine significant differences between neonatal and adult mice: (i) in the percentage of time spent in wakefulness, NREM, or REM sleep states, (ii) and in the relative band power of EEG in delta (δ), theta (θ), and beta (β) during behavioral states. In case of significant differences, post-hoc analyses were performed by Tukey’s test. *p*-values < 0.05 were considered statistically significant. Statistical analyses were performed with SigmaPlot (v. 12.0, Systat Software, Erkrath, Germany).

## 3. Results

**Figure 1:**
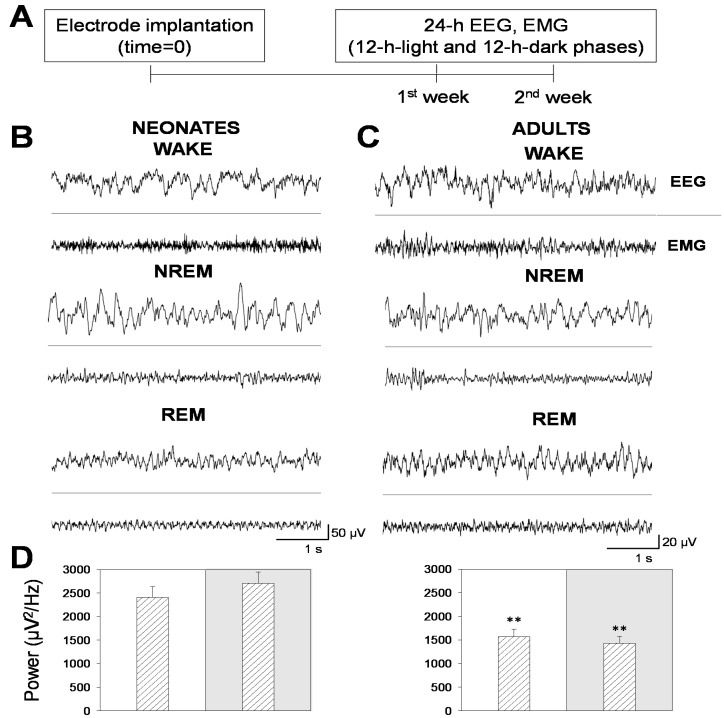
Electroencephalogram (EEG) and electromyogram (EMG) recordings in neonatal and adult mice. (**A**) Experimental design for EEG and EMG longitudinal recordings in mice. Following electrode implantation and a post-surgical recovery period, mice were recorded during two consecutive weeks in continuous 48 h recording sessions per week. (**B**,**C**) Representative 5 s EEG and EMG traces of behavioral states (wakefulness, non-rapid eye movement (NREM), and rapid eye movement (REM) sleep in neonatal (left) and adult (right) mice. (**D**) Bars represent the total power within the 0–100 Hz. frequencies in neonatal (left panel) and adult (right panel) mice. White or gray backgrounds represent 12 h-light or 12 h-dark phase, respectively. The graphs below show the total power as a function of the recording time in a 24 h period. Data shown as mean ± SEM of either 18 or 27 recordings retrieved from 6 neonatal or 11 adult mice, respectively. Asterisks denote significant differences between neonatal and adult mice: ** *p* < 0.01; Unpaired *t*-test.

In this work, we analyzed the sleep–wake cycle and characterized the patterns of electrical activity in late neonatal mice, an age not previously characterized [13]. We acquired longitudinal EEG and EMG recordings in 24 h sessions, split into 12 h-light and 12 h-dark phases, during two consecutive days per week, for two weeks following the experimental scheme illustrated in Figure 1A. Behavioral states (wakefulness and NREM and REM sleep) were determined by spectral analysis of EEG and EMG recordings in neonatal (Figure 1B) and adult (Figure 1C) mice. Note that neonatal mice showed signal amplitudes that were 2.5 times greater than adult mice, which translated into higher EEG total power (Figure 1D). 

**Figure 2:**
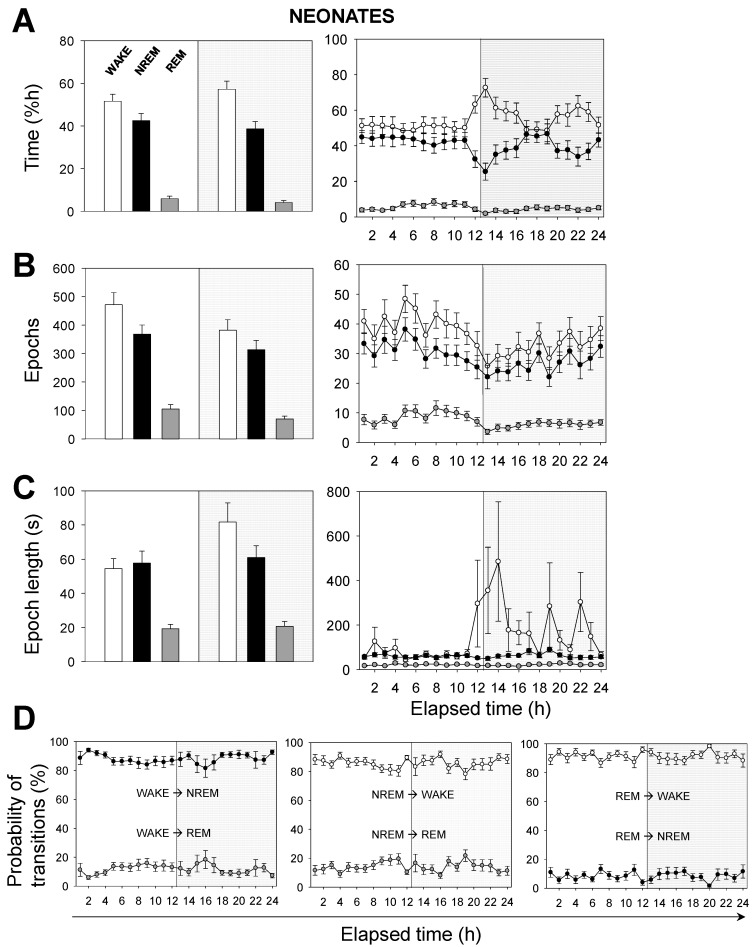
Sleep–wake characterization in neonatal mice. (**A**) Percentage of time spent, (**B**) total number of epochs, and (**C**) epoch length. White, black, or gray bars represent wakefulness, NREM, or REM sleep states, respectively. Parameters in (**A**–**C**) are calculated during a total of 12 h-light (white background) or 12 h-dark (gray background) phases (left panels) or across the recording time within the 24 h period (right panels). (**D**) Probability of behavioral state transitions for every hour during the recording session (1–24 h.). Left panel: From wakefulness into NREM and REM sleep. Middle panel: From NREM sleep into wakefulness and REM sleep. Right panel: From REM sleep into wakefulness and NREM sleep. White, black, or gray circles represent wakefulness, NREM, or REM sleep states, respectively. Data shown as mean ± SEM of 18 recordings retrieved from 6 neonatal mice.

**Figure 3:**
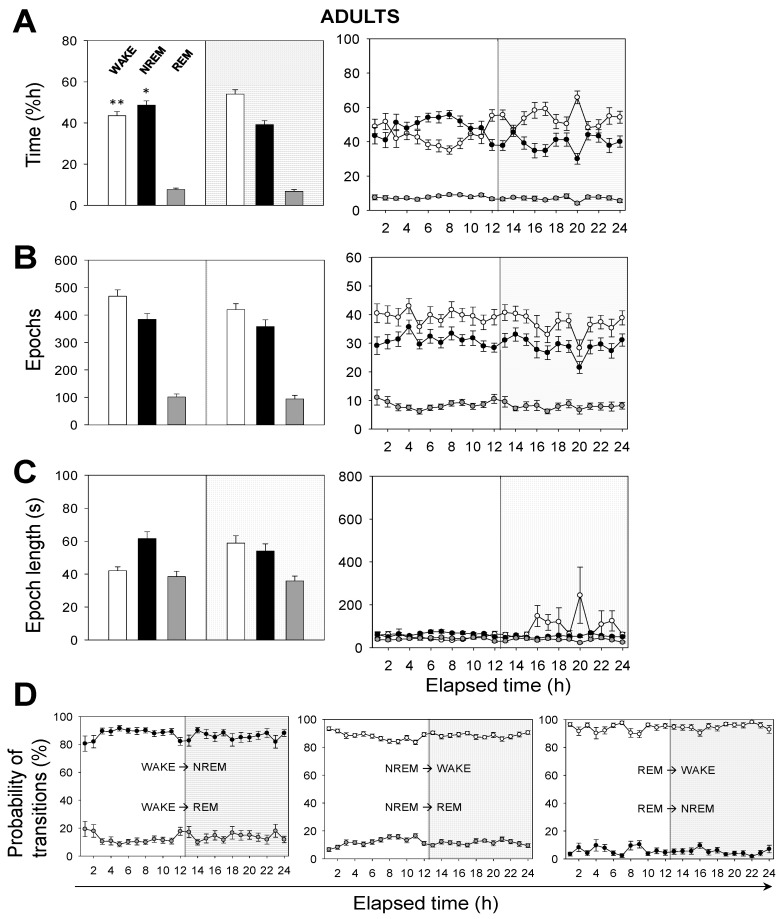
Sleep–wake characterization in adult mice. (**A**) Percentage of time spent, (**B**) total number of epochs, and (**C**) epoch length. White, black, or gray bars represent wakefulness, NREM, or REM sleep states, respectively. Parameters in (**A**–**C**) are calculated during a total of 12 h-light (white background) or 12 h-dark (gray background) phases (left panels) or across the recording time within the 24 h period (right panels). (**D**) Probability of behavioral state transitions for every hour during the recording session (1–24 h.). Left panel: From wakefulness into NREM and REM sleep. Middle panel: From NREM sleep into wakefulness and REM sleep. Right panel: from REM sleep into wakefulness and NREM sleep. White, black, or gray circles represent wakefulness, NREM, or REM sleep states, respectively. Data shown as mean ± SEM of 27 recordings retrieved from 11 adult mice. Asterisks denote significant differences between neonatal and adult mice: * *p* < 0.05; ** *p* < 0.01; Two-way ANOVA and Tukey’s test for post-hoc analysis.

Next, we performed a time analysis of the sleep–wake cycle. We confirmed that the percentage of time spent in wakefulness was higher in neonatal than in adult mice, and the opposite occurred in NREM sleep (Figure 2A vs. Figure 3A). Remarkably, we found no significant differences, between age groups, in the percentage of time spent in REM sleep, which was independent of the light/dark cycle (Figure 2A vs. Figure 3A).

Hourly data trends during the recording session confirmed that both neonatal and adult mice, overall, (i) spent more time sleeping during the 12 h-light phase, and (ii) spent more time awake during the 12 h-dark phase (Figure 2A vs. Figure 3A). This behavior is expected due to rodents being prey and avoiding diurnal predators. Note that the trends slightly shift a few hours before the phase transitions, meaning that the circadian cycle is synchronized with the external conditions. To clarify these results we computed the number of epochs and the epoch lengths that corresponded to each behavioral state. In neonates (Figure 2B,C), the number of epochs for each behavioral state, especially for wakefulness, decreased during the 12 h-dark phase. Wakefulness epochs were longer at nighttime than they were at daytime, while those of NREM and REM sleep remained steady. In adults, the number of epochs for each behavioral state tended to reduce during the recording session, showing slightly higher values at daytime than those at nighttime (Figure 3B,C). Wakefulness epochs were longer at nighttime than they were at daytime, contrary to NREM sleep epochs. Note that NREM sleep epochs were prolonged at daytime and were only slightly shorter at nighttime. Collectively, comparing neonatal and adult mice, we noticed that neonatal mice showed longer wakefulness epochs, especially at nighttime, and shorter REM sleep epochs, overall (Figure 2C and Figure 3C). As for the number of epochs, neonatal mice showed a slight reduction for each behavioral state, especially at nighttime, correlating with the phase transition (Figure 2B and Figure 3B).

The probability of transitions in behavioral states was similar in both neonatal and adult mice (Figure 2D and Figure 3D). Hourly data trends during the recording session showed mid-phase (6 h elapsed time) shifts, which confirmed the higher probability of transitions from NREM sleep to wakefulness than from NREM to REM sleep (86% vs. 14%, for neonates), from wakefulness to NREM sleep than from wakefulness to REM sleep (88% vs. 12%), and from REM sleep to wakefulness than from REM to NREM sleep (91% vs. 9%). That is, transitions between behavioral states in mice shift in periods of 6 h from the start or end of the light phases, reaching mid-phase plateaus. Thus, the sleep–wake cycle slowly adapts to the external changes and prepares for the next state.

**Figure 4:**
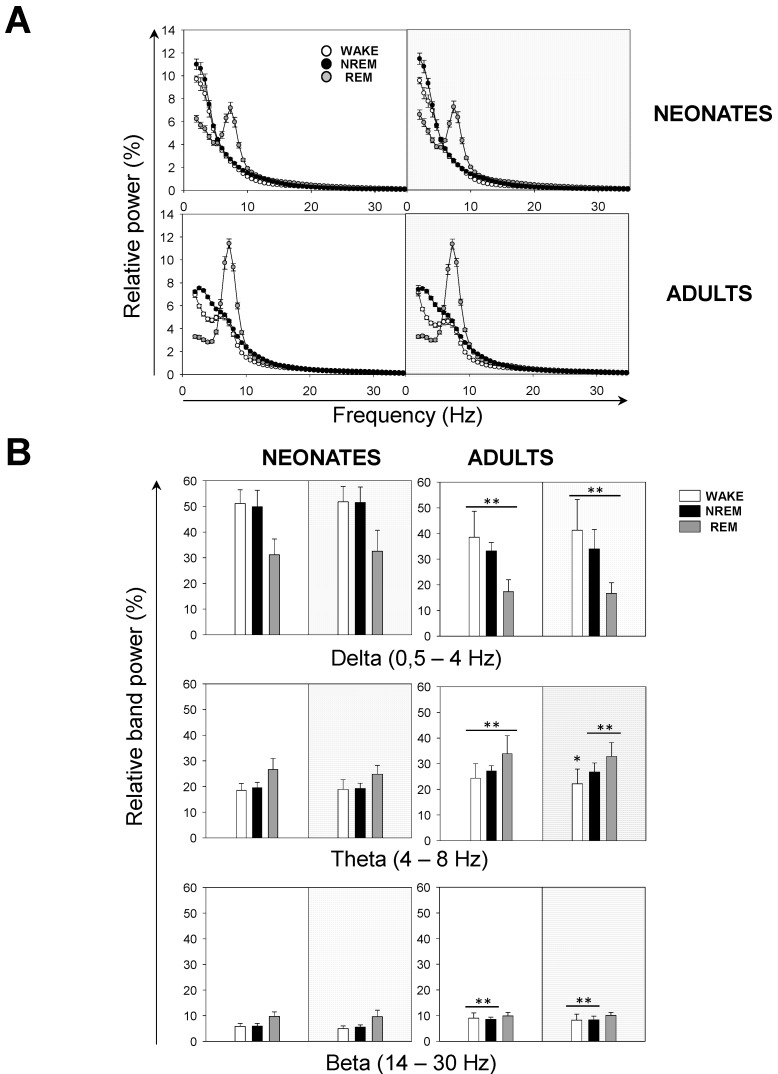
Sleep–wake EEG spectra during behavioral states in neonatal and adult mice. (**A**) Influence of light/dark cycle in the relative power of EEG during behavioral states in neonatal (top panels) and adult (bottom panels) mice. (**B**) Relative band power of EEG in delta (δ), theta (θ), and beta (β) during behavioral states. Data shown as mean ± SEM of either 18 or 27 recordings retrieved from 6 neonatal or 11 adult mice, respectively. Asterisks denote significant differences between neonatal and adult mice: * *p* < 0.05; ** *p* < 0.01; Two-way ANOVA and Tukey’s test for post-hoc analysis.

Next, we performed a spectral analysis of the sleep–wake cycle in order to examine the behavior of neonatal and adult mice with respect to the patterns of brain rhythmicity. We found brain maturation signs associated with the EEG relative power within the 0–35 Hz band (Figure 4A). Adult mice showed the expected trends for relative power (Figure 4A): wakefulness was predominantly characterized by δ rhythms and a smaller θ component, NREM sleep showed a predominant δ component, and REM sleep featured a strong θ component. The differences between neonatal and adult mice were the following (Figure 4A): in neonatal age, each behavioral state showed (i) a stronger δ component, especially during wakefulness and NREM sleep, and (ii) a reduced θ component, especially during wakefulness and REM sleep. In order to evaluate these differences more closely, we computed the behavioral-state-dependent relative band power for neonatal and adult mice (Figure 4B). There were no remarkable differences in the relative band power between both light phases for any frequency band within either age group. Within the delta (δ) frequency band, neonates were characterized by a greater relative power than adults for each behavioral state. Wakefulness showed the greatest relative power, followed closely by that of NREM sleep. Within the theta (θ) frequency band, we found the opposite pattern: adults were characterized by a higher relative power than neonates for each behavioral state. REM sleep showed the greatest relative power. Within α (and β, during wakefulness and NREM sleep) frequency bands, we concluded the same as for the θ frequency band upon the neonate–adult pattern, but with overall smaller relative band powers for each behavioral state (Figure 4B and Figure S1). Although the contribution of sigma (σ) and γ frequency bands was very small, noticeable intergroup differences showed a stronger σ component during NREM, and a stronger γ component during wakefulness for adults (Figure S1). Overall, we concluded that neonatal mice showed an increased relative δ band power, and a decreased relative θ (and β, during wakefulness and NREM sleep) band power, in comparison to adult mice.

## 4. Discussion

A deeper understanding of sleep during the transition from early to adult ages might help us to better understand how cerebral rhythms change with brain development. This knowledge is key to guide our research with animal models for specific biomarkers that might result impaired after injury and neurodegeneration. Previous studies have characterized the sleep–wake cycle in mice at very early ages (<3 weeks) revealing that behavioral sleep–wake states were not reflected in EEG until the 12th postnatal day [3,14]. To our knowledge, no specific analysis of cerebral rhythms has been performed in late neonatal mice (4 weeks), equivalent to pediatric ages [13].

Neonatal mammals spend more time in REM sleep, a fact probably linked to the development of neuronal circuitry [3,4,5,15]. In humans, early neonates spend 50% of their time in REM sleep at birth, 40% at three–five months and 30% at the age of 1–2 years [16]. In our study, we did not detect differences in the time spent in REM sleep between neonatal and adult mice. This is probably due the time point of EEG analysis (4 weeks), which probably reflects maturity sufficiently for REM sleep to not be as dominant as in early neonatal mice. By contrast, our results showed a behavioral-state-independent reduction in θ band frequencies in late neonatal mice with respect to adult mice. While the EEG analysis differed, our results are in agreement with [14,17] for neonates and adults, respectively; and are consistent with the hypothesis that θ band components develop at a later, juvenile stage, which is similar to humans [16].

It has been previously described that total power increases with maturation in early neonatal mice, but this was not compared to adult mice [14]. Our results point to a greater total power in late neonatal mice than in adult mice. That is, total power would be very low in infant mice. In early neonatal mice, it would steadily increase up to the late neonatal stage. At this time, the total power would be very high. Eventually, at a later, juvenile stage, the total power would drop to typical adult amplitudes. We hypothesize that aging would also have an effect on total power so that the amplitudes shown in elderly mice might differ to those in adults. This is supported by Zappasodi et al. [18] in a study carried out in healthy humans, which found age-dependent changes in EEG complexity, and band powers. Namely, they showed a parabolic evolution of fractal dimension (and therefore, band powers) with aging.

Four main experimental findings can be extracted from our study. Late neonatal mice (i) spent more time in wakefulness and less time in NREM sleep than adult mice, (ii) showed a reduced relative θ band power compared to adult mice, as well as an increased relative δ band power in neonatal mice compared to adult mice, (iii) showed a reduced relative band power in β during wakefulness and NREM sleep, and (iv) showed a higher total power over all frequencies. Our conclusions agree with those presented by Rensing et al. [14] regarding the relative δ and θ band power, and by Zappasodi et al. [18], regarding the relative β band power, and provide additional insights into late neonatal development and a comparison with adult features.

In advanced phases of brain neurodegeneration EEG and sleep abnormalities might serve as diagnostic features, and may aid in predicting functional outcomes of disease progression. Two recent epidemiologic studies, one longitudinal and the other cross-sectional, found that (i) midlife insomnia is associated with a higher risk of late-life dementia [19], and (ii) insomnia in individuals at risk of AD is associated with both cognitive and brain structural patterns, respectively [20]; another study also found that patients with primary insomnia show an increased sigma and beta band power during NREM sleep [21]. It could as well be applied to mental disorders such as the borderline personality disorder, which is known to present an increased delta band power during NREM sleep [22]. The data presented here for adult mice might be compared with the human sleep reference data provided by Gabryelska et al. [23] and Hertenstein et al. [24] for research on mental and physical health. Considering neurodegenerative models and the predictability expected from them, the analysis of EEG and cerebral rhythms in non-modified mice from late neonatal to adult can help to identify specific development patterns during brain maturation that might be impaired at preclinical and prodromal diagnosis, decades before clinical neurodegeneration manifests.

## Data Availability

The following are available online at https://doi.org/10.17605/OSF.IO/6FWYE, Dataset S1: EEG and EMG recordings of late neonatal (*n* = 18; aged 30 days), and adult (*n* = 27; aged 3 months) male C57BL/6 mice, Dataset S2: Sleep–wake cycle analysis (1: REM; 2: WAKE; 3: NREM; 4: UNDEFINED) of late neonatal (*n* = 18; aged 30 days), and adult (*n* = 27; aged 3 months) male C57BL/6 mice.

## Supplementary Materials

The following are available online at https://www.mdpi.com/2076-3425/11/3/298/s1, Figure S1: Relative band power of EEG during behavioral states in neonatal and adult mice. Dataset S1: EEG and EMG recordings of late neonatal (*n* = 18; aged 30 days), and adult (*n* = 27; aged 3 months) male C57BL/6 mice, Dataset S2: Sleep–wake cycle analysis (1: REM; 2: WAKE; 3: NREM; 4: UNDEFINED) of late neonatal (*n* = 18; aged 30 days), and adult (*n* = 27; aged 3 months) male C57BL/6 mice.
