# Supplementary material for: Sleep–Wake Cycle and EEG–Based Biomarkers during Late Neonate to Adult Transition
Source: Brain Sci. 2021 Feb 27;11(3):298. doi: 10.3390/brainsci11030298 (PMC7996792; doi:10.3390/brainsci11030298)
Supplement: Supplementary file 1 [file brainsci-11-00298-s001.pdf]

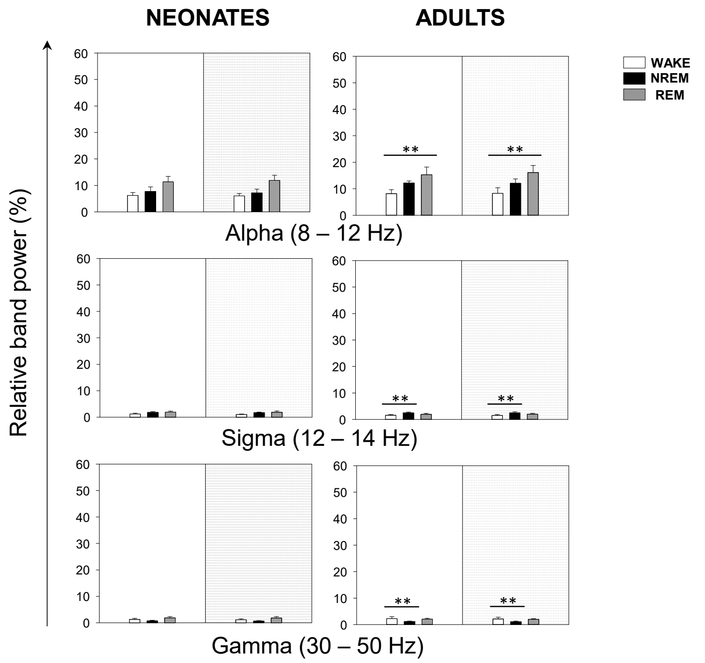

Figure S1. Relative band power of EEG during behavioral states in neonatal and adult mice. Relative band power of EEG in alpha (α), sigma (σ), and gamma (γ) during behavioral states. Data shown as mean ± SEM of either 18 or 27 recordings retrieved from 6 neonatal or 11 adult mice, respectively. Asterisks denote significant differences between neonatal and adult mice: ** $p < 0.01$; Two-way ANOVA and Tukey's test for post-hoc analysis.
